# Spinal cord stimulation combined with exercise in patients diagnosed with persistent spinal pain syndrome. Study protocol for a randomized control trial

**DOI:** 10.1371/journal.pone.0309935

**Published:** 2024-10-31

**Authors:** J. Vicente-Mampel, F. Falaguera-Vera, D. Sánchez-Poveda, F. Hernández-Zaballos, M. Martinez-Soler, P. Blanco-Giménez, F. J. Sanchez-Montero

**Affiliations:** 1 Department of Physiotherapy, School of Medicine and Health Science, Catholic University of Valencia, Torrent, Valencia, Spain; 2 Specialist Physician, Anesthesiology Service, Pain Unit, Complejo Asistencial Universitario de Salamanca, Salamanca, Spain (CAUSA); Adnan Menderes Universitesi, TÜRKIYE

## Abstract

**Introduction:**

Administration of spinal cord stimulation to individuals with PSPS-T1/2 may induce supraspinal descending activation. Similarly, exercise is recognized as a fundamental aspect of spinal pain management. Studies have demonstrated its impact on neurophysiological factors, including the release of spinal and supraspinal beta-endorphins, which activate μ-opioid receptors. Therefore, the purpose of this study will be to examine the effect of SCS in combination with lumbo-pelvic stability core training on perceived low back pain, quality of life and disability in PSPS-T2 patients.

**Methods/Materials:**

A double-blind randomized clinical trial (RCT) has been designed. All participants will be randomized from a pre-set sequence. The intervention design has been elaborated from the CONSORT guidelines. This study has been registered at Clinicaltrial.gov (NCT06272539). Sample size was calculated using G Power® Sample size software (University of Düsseldorf). The calculation was based on a moderate effect size of 0.7 (partial η2 = 0.70, α = .05, power = 0.95), resulting in a total of 40 patients. Assuming a 30% dropout rate, 52 participants will be recruited in total. Two sessions per week will be scheduled for 8 weeks with a total of 16 sessions. Each work session will have a duration of 60 minutes. The exercise will be adapted according to the phases based on the results already published, limiting in each phase the degrees of flexion and extension of the spine to avoid the risk of electrode migration. Primary outcomes will be functionality, satisfaction, strength, psychosocial variables, quality of life and pain perception.

## 1. Background

Chronic spinal pain is characterized by persistent pain that is not always accompanied by structural abnormalities, leading to a discordance between the two [1]. A common clinical presentation of this type of pain is failed back surgery syndrome (FBSS) [2]. However, diagnostic labels for this condition have been considered inadequate, misleading, and potentially troublesome [3]. As an alternative, the term persistent spinal pain syndrome (PSPS) [4] has gained interest due to its high prevalence and recurrence in the clinical practice of pain management specialists [5]. Consequently, new nomenclature, PSPS-T1 and -T2, is now routinely used in publications [6]. The primary symptoms of chronic spinal pain include lower back pain, severe disability, poor quality of life, and high unemployment rates [7]. Additionally, the heterogeneity of these patients complicates diagnosis and evaluates the clinical effect of each treatment modality [8]. This may be due to the existence of peripheral and central mechanisms that trigger the state of the central nervous system [9].

The PSPS-T2 condition has the potential to affect a substantial number of people in the general population, with several factors related to the nervous system’s neuroplasticity. The progression and adaptation of the condition may be influenced by biological diversity, as well as psychological and social factors [10]. To aid in muscle recovery during rehabilitation, advanced treatments, including the use of neuromodulatory strategies, are employed [11]. Therapeutic approaches for PSPS-T2 vary and include surgical reintervention, pharmacological treatments, and more conservative methods that focus on exercise and behavioral therapies [12,13]. Spinal cord stimulation (SCS) is one such intervention, with a medium-term effect that can last up to 2 or 3 years [14] although several studies suggest that long-term outcomes are favourable with regard to pain relief among individuals with predominant radicular pain [15]. SCS has been shown to alleviate pain and reduce analgesic consumption, and improve quality of life [16]. However, the programming of SCS may modify glial signaling through the modulation of neuronal and glial cell activation in patients, and the effects of SCS on patients with persistent spinal syndrome are yet to be determined [17]. Nevertheless, the conditioned modulation of pain may be associated with the effect of SCS [18].

In terms of published findings, there is a remarkably low quality for the majority of the use of SCS for treating persistent pain [19]. The intricacy of pain necessitates healthcare professionals to propose a multidisciplinary approach [20]. To this end, a patient-centered approach has recently been regarded as a crucial component for individuals with persistent pain [21]. Exercise therapy, in particular, is considered the fundamental aspect of spinal pain treatment [22]. Research indicates that motor control and spinal stabilization exercises, as opposed to surgical and pharmacological interventions, are more effective in reducing pain and disability for patients with spinal pain [23,24]. Recent observations have emphasized the significance of muscle inhibition as a trigger for loss of function and pain in patients with chronic low back pain [25]. In addition to its biomechanical effects, exercise treatment is an intervention that supports self-management of care [26] by reinforcing the utilization of strategies to enhance biopsychosocial beliefs [27].

The application of integrated interventions may lead to enhanced results in both the short-term and medium-term periods [28]. Combining SCS therapy with physical exercise may not only improve outcomes, but it may also result in a higher number of patients experiencing sustained benefits. However, it is important to consider the potential adverse events that may occur when implementing spinal cord stimulation (SCS) and exercise together, such as electrode migration. Approximately one in ten patients who receive SCS implants experience lead migration [29]. Additionally, the high incidence of lead displacements (3.07%) should not be overlooked [30]. According to the postoperative treatment guidelines, patients are advised to refrain from engaging in any form of exercise or assuming positions that involve the end range of motion in the lumbar spine for a period of two months following the operation. This precautionary measure is necessary as it can potentially result in the migration of the implanted electrode. It is essential to allow sufficient time for the surrounding tissues to undergo fibrosis and securely anchor the electrode in place. Patients with PSPS T2 may have protective attitudes influenced by fear of pain due to movement (kinesiophobia), which can exacerbate patient deconditioning [31]. Therefore, treatment should focus on enhancing the patient’s perceptions and beliefs about movement and addressing not only mechanical and functional parameters but also improving the overall experience [32]. The use of both treatment techniques (SCS/exercise) has resulted in moderate-quality evidence, presenting low-effect measurements when applied in isolation [33]. Furthermore, implementing an exercise protocol that minimizes the risk of adverse effects is crucial. Designing an exercise program based on the range of motion of the spine may be particularly beneficial in avoiding adverse effects on SCS. This study aimed to evaluate the effect of combined treatment with SCS and exercise focused on neuromuscular control of CORE in comparison with isolated treatment with SCS on functionality, pain perception, psychosocial variables, patient satisfaction, adverse effects, and quality of life in patients diagnosed with PSPS-T2. Based on these recommendations, the hypothesis proposed in this study aimed to capitalize on the analgesic effect of posterior cord stimulation during a window of opportunity while the patient underwent re-education through neuromuscular and core control exercises. The integration of these two treatment techniques is expected to yield superior effects compared to the application of a single treatment in isolation.

## 2. Materials and methods

### 2.1 Study design

**Fig 1:**
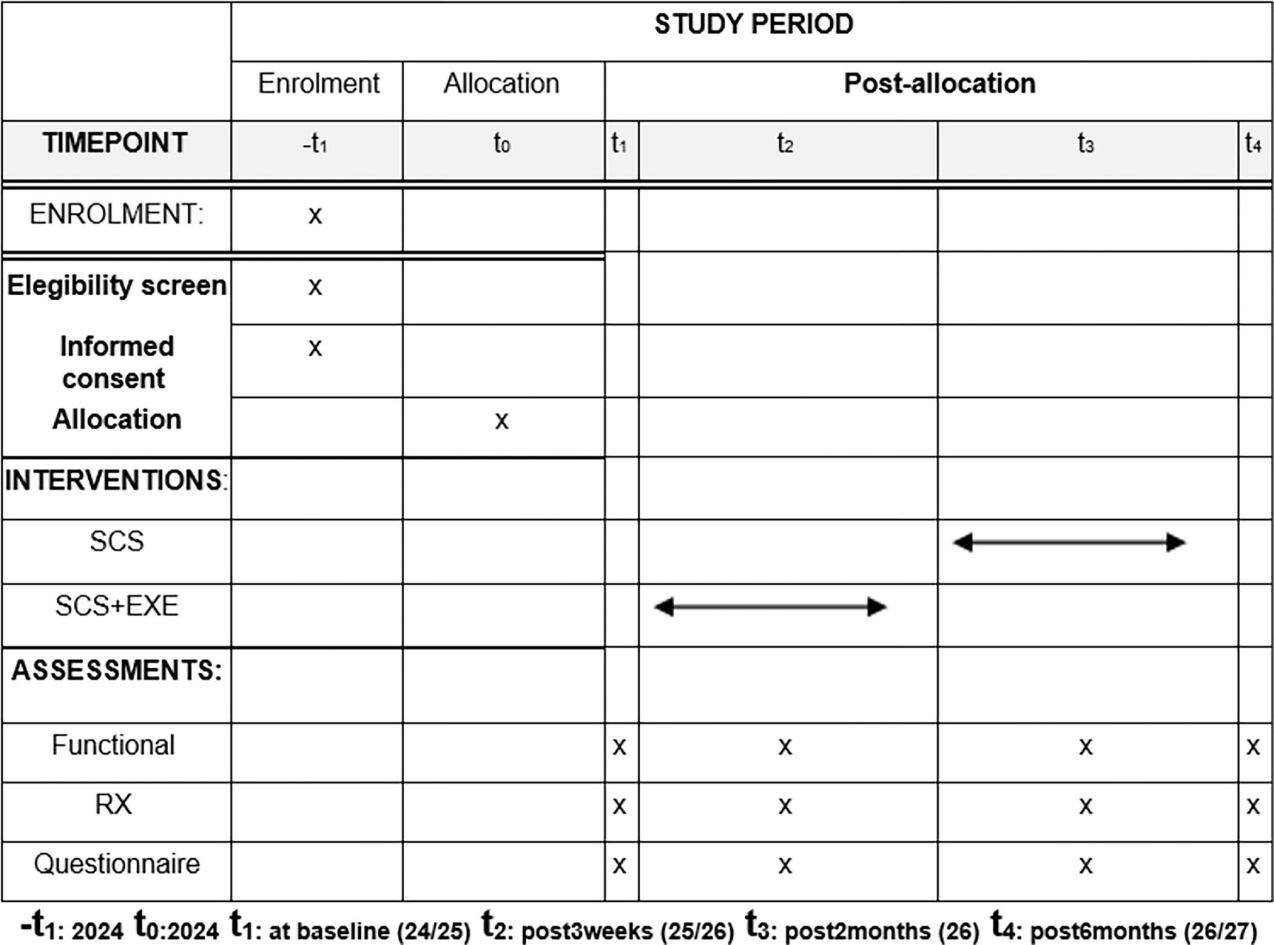
Execution schedule–recruitment, intervention, and reassessment.

This study was designed as a simple-blinded, comparative longitudinal, and prospective randomized controlled trial (RCT). The protocol was written according to the SPIRIT statement, increasing its transparency and completeness (Fig 1) [34]. The findings will be reported following the TIDieR checklist [35].

This study will be a research protocol for two groups: SCS and core and control motor exercises with an allocation ratio of 1:1. Data will be collected from April 2024 to January 2026. All participants will sign an informed consent form, prepared in accordance with the ethical guidelines of the Helsinki Declaration [29]. The study protocol was approved by the Ethics Committee on January 24, 2024 (ID: 2023 10 1435). This study was prospectively registered in the Clinical Trials (Registration number: NCT06272539) (Supplementary file 1)

### 2.2 Informed consent

After assessing the patient and obtaining pertinent information, individuals who willingly express their interest in participating in the study and have signed the necessary informed consent forms will be chosen based on predetermined eligibility criteria. These selected participants will then be randomly assigned.

### 2.3 Study population

The participants will be patients diagnosed with spinal pain syndrome who are undergoing SCS at the Pain Unit at the Hospital of Salamanca. The participants will be selected according to the compliance criteria established by the criteria established by the guidance Neurostimulation Appropriateness Consensus Committee (NACC) of neurostimulation practice [36]. All patients will be informed of all the study procedures, and those of interest will be provided with a brief introduction to the selection process.

### 2.4 Inclusion and exclusion criteria

The eligibility inclusion criteria will be as follows: diagnosis of PSPS-T2 with leg and back pain; ii) patients older than 18 years; iii) ≥6 months with pain and iv) VAS score >7. Exclusion criteria were: (i) previous surgeries in the abdominal area, (ii) pregnancy or lactation, (iii) severe fractures or pathologies, (iv) spinal structural deformity, and (vi) neurologic or psychiatric issues.

### 2.5 Procedure

**Fig 2:**
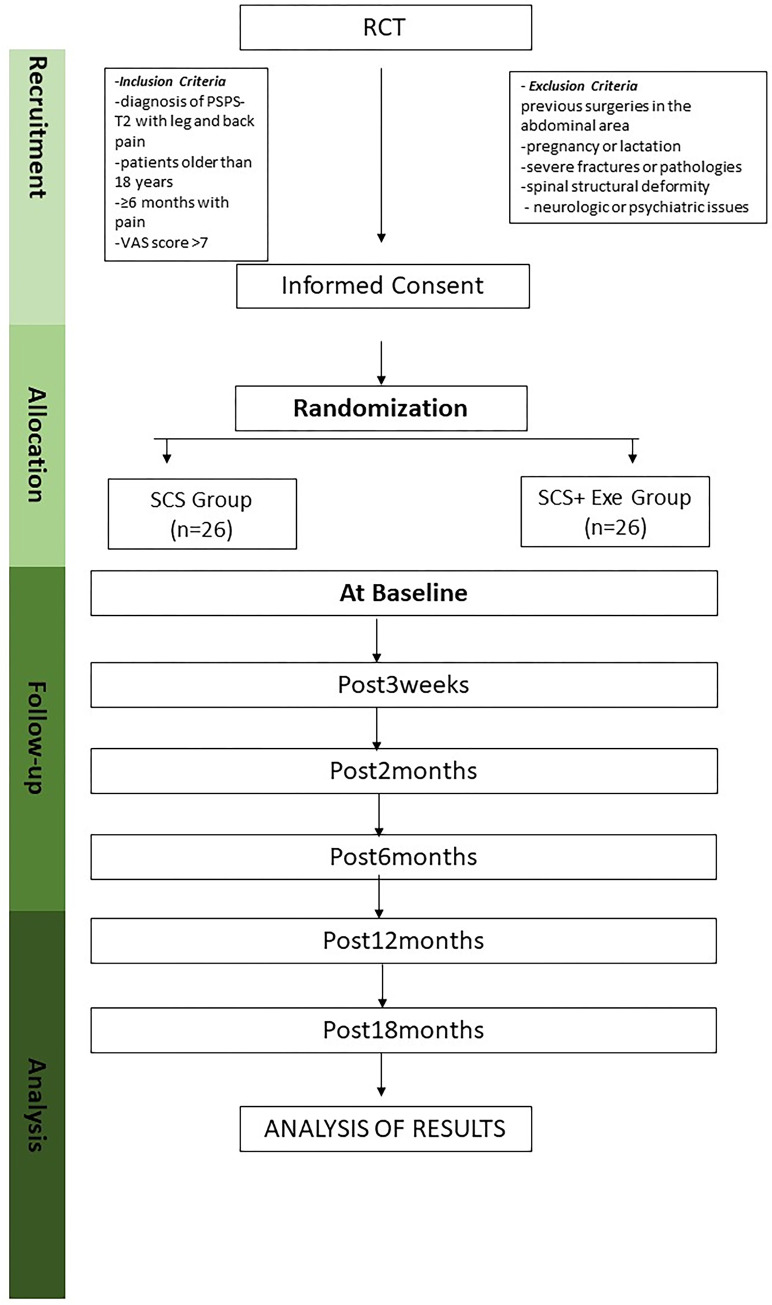
Randomization flow chart and protocol for intervention measurements.

Once the groups have been established, there will be six sample collections: Pre, Post3weeks, Post2months, Post6months, Post12months and Post18months (Fig 2). Patients will be introduced to the CORE neuromuscular and control motor exercise protocol after surgery.

## 3. Randomization and blinding

By doing so, we aim to eliminate or reduce potential biases due to the order of patients and any transference effects that might occur if one profile clinical patient will influence the performance in several evaluations. All individuals in the treatment cohort will be overseen by two physiotherapists with extensive experience (>10 years). One physiotherapist will administer the interventions for all two groups, while the other will perform the evaluations, ensuring that the second physiotherapist remains blinded to the evaluated group. An unbiased outcome ascertainment will be ensured by an independent researcher who will create a table of random numbers using an Excel formula to blind data collectors and outcome adjudicators. A block randomization design with block sizes of 4 or 8 will be applied to ensure equal distribution of participants across each group. The randomization sequence will be secured on a USB drive and stored under lock and key by an independent researcher, accessible only when necessary. A single-blind design will be employed due to the impossibility of blinded participants and the treating physiotherapist for exercise intervention. Specifically, each patient will complete a sequence of allocation in the order of A, B, B, A, where ’A’ represents SCS treatment isolated, and ’B’ denotes combined treatment. Subsequently, the average of the two ’A’ conditions was calculated, and the same process was applied for the ’B’ conditions.

## 4. Sample size

The sample size will be estimated using GPower® software (Franz Faul, Universität Kiel, Kiel, Germany), version 3.1.9.2. As there are no similar studies allowing for the calculation of sample size based on reference means and standard deviations, a comparison will be made using the difference between two dependent means of 1.95 normal populations, assuming equal standard deviations of 3,2 according to the results obtained from a prior study [37]. Thus, the calculation will be based on the primary outcome of "Disability" and will consider an effect size of 0.70, a power of 0.95 for ANOVA repeated measures, and an alpha error of 0.05. A total of 40 participants (20 subjects per group) will be included in this study. Moreover, considering the probability of loss during follow-up (30%), 12 more participants considering dropout (6 participants per group) will be included, resulting in a total of 52 participants. The possibility of increasing the number of subjects will be explored in case of insufficient statistical power to reach these predefined levels. In the event of dropouts, noncompliance, or absence of results, an intention-to-treat analysis will be conducted.

### 4.1 Interventions

Patients included in the study will be randomised to receive:

#### 4.1.1 Lumbo-pelvic core stability training program combined with motor control exercises

The experimental group will undertake a lumbo-pelvic core stability training program that incorporates motor control exercises through specific therapeutic interventions at the lumbopelvic center, along with neurostimulation treatment. This intervention plan was designed in accordance with the principles established by Falla et al. [22]. The exercises in each phase are specifically designed to limit the degree of flexion/extension and lumbar traction. Two weekly sessions, each lasting 60 minutes and spanning eight weeks, will constitute a total of 24 sessions. A certified physiotherapist with at least 10 years of clinical experience will administer this treatment.

*Phase 1 (muscular activation)*

In the initial phase of the study, all participants will be instructed on how to engage their abdominal muscles effectively. Feedback and ultrasound imaging will be utilized to ensure proper training of the participants. This phase will last for 15 days, comprising of four sessions, with the primary objective of achieving voluntary neuromuscular control. There is no need for flexion-extension movements in the spine exceeding 65 degrees in both directions during this phase, thus eliminating the risk of electrode migration. Participants will learn to engage their abdominal muscles through techniques such as rib breathing and forced exhalation, which will result in the contraction of the internal obliques, the activation of the multifidus lumbar, and the easing of the abdominal transverse.

The exercises that will be performed during this phase are detailed in Tables 1 and 2.

**Table 1:** Phase 1 exercise schedule for the first and third sessions.

	Sessions 1 and 3						
	Exercise	Series	Repetitions	Intensity	Duration	Break	Effort
**Block 1**	*Breathing dissociation*	4	15–20	2/10	-	30”	Minimal
	*Iso hip adduction + breathing dissociation*	4	10–12	3/10	-	30”	Minimal
	*Iso horizontal push + breathing dissociation*	4	6–8	6/10	-	30”	Low
**Block 2**	*Hollowing + FB ultrasound*	3	5	4/10	-	30”	Low
	*Iso pelvic retroversion + FB stabilizer + hollowing*	3	12–15	4/10	-	30”	Low
	*Bracing + FB ultrasound*	3	5	7-8/10	-	30”	Moderate
**Block 3**	*Hip abduction in dead bug position with feet against the wall*	4	20–25	9/10	1,3,3	-	High
	*Internal rotation and hip rotation from dead bug position + forced exhalation*	4	5x5”	9/10	-	-	High
	*Iso holds anti extensor facing up*	4	5x5”	7/10	-	120”	Moderate

**Table 2:** Phase 1 exercise schedule for the second and fourth sessions.

	Sessions 2 and 4						
	Exercise	Series	Repetitions	Intensity	Duration	Break	Effort
**Block 1**	*Hollowing + FB ultrasound*	3	5	4-5/10	-	30”	Low
	*Iso pelvic retroversion + FB stabilizer + hollowing*	3	12–15	4/10	-	30”	Low
	*Bracing + FB ultrasound*	3	5	7-8/10	-	30”	Moderate
**Block 2**	*Iso hip extension push from 90° flexion standing*	4	5x5”	9/10	-	-	Low
	*Bracing + press pallof sitting horizontal with asymmetric feet*	4	8–10	5/10	1,3,2	30”	Low
	*Bracing + press pallof a stride*	4	8–10	5/10	1,3,2	30”	Moderate
**Block 3**	*Monsters walk for medial gluteus guided in parallel*	4	15/side	8/10	1,3,3	30”	Moderate
	*Isotonic clamshell*	4	15–25	9/10	-	-	High
	*Iso holds against rotation facing up in crossed position with hip at 90° and external knees unbalancing + bracing*	4	5x5”	8/10	-	60”	Moderate

*Phase 2A Posture/ alignment*

The second phase of treatment will span from day 16 to day 37 after the intervention. This phase aims to focus on exercises that target the deep spinous-transverse muscles (multifidus), which serve as essential stabilizers for individual spinal segments. As patients have already improved their neuromuscular control during phase 1, the emphasis shifts towards initiating muscle recovery. In this phase, exercises will involve a greater flexion range starting from 65 degrees, similar to phase 1, while the extension range will be extended to 85 degrees. The extension movement of the spine is considered safe as it does not increase neural tension, utilizing the posterior pillar of the facet joints instead. During the 21-day treatment period, patients will attend six monitoring sessions and perform exercises at home safely, following the instructions provided. The exercise progression will be tailored to each patient’s ability based on their pain level. Exercises will be performed from series 1 to 3, with 8 to 15 repetitions, and isometric contractions lasting between 5 and 10 seconds. Breaks between series will last 30 seconds, while breaks between exercises will be 2 to 3 minutes [38].

The exercises for this phase are listed in Tables 3–5.

**Table 3:** Phase 2A exercise schedule for the first, third and fifth sessions.

	Sessions 1, 3 and 5						
	Exercises	Series	Repetitions	Intensity	Duration	Break	Effort
**Block 1**	*Bracing + FB ultrasound*	3	10	7-8/10	-	30”	Low
	*Iso holds anti extensor facing up*	3	8x5”	7/10	-	30”	Low
	*Iso hold autorotation facing up*, *in cross position with hip at 90° and external knees unbalancing+ bracing*	3	10x5”	8/10	-	30”	Moderate
**Block 2**	*Ascendant row for dorsal external extension a stride with blocked lumbar*	3	12–15	8/10	1,3,3	-	Moderate
	*Dorsal inclination with iso push from knight position (closed side) + exhalation*	3	5x5”	8-9/10	-	-	Moderate
	*Iso push hip extension standing in one leg over asymmetric ground + soft hinge with KB in goblet squat*	3	10	7/10	-	120”	Low
**Block 3**	*Isotonic clamshell*	4	15–25	9/10	-	-	High
	*Bracing + vertical press pallof a stride*	4	8–10	5/10	1,3,2	-	Low
	*Monsters walk for medial gluteus guided in parallel*	4	15/side	9/10	1,3,3	120”	High

**Table 4:** Phase 2A exercise schedule for the second, fourth and sixth sessions.

	Sessions 2, 4 and 6						
	Exercise	Series	Repetitions	Intensity	Duration	Break	Effort
**Block 1**	*Dead bug with support + iso holds from anti extension arms + bracing*	3	6x5”	8/10	-	30”	Moderate
	*Dead bug with switch changing support + iso hold antiextension arms + bracing*	3	10	8/10	-	30”	Moderate
	*Iso hold antirotation facing up*, *cross position with hip at 90° and external instability from knees + bracing*	3	10x5”	8/10	-	30”	Moderate
**Block 2**	*Press pallof horizontal standing over asymmetric ground + bracing*	3	12–15	8/10	1,3,3	-	Moderate
	*Iso push hip extension at 30°*, *standing up with blocked core*	3	5x5”	8-9/10	-	30”	Moderate
	*Standing in one leg iso push over asymmetric ground + soft hinge in goblet squat*	3	10	7/10	-	120”	Low
**Block 3**	*Hip abduction in stable sitting*, *iso push creating speed peaks and contraction intensity*	4	5x7”	9/10	-	30”	High
	*Vertical press pallof sitting with asymmetric legs from 130° arms flexion*	4	8	7/10	-	30”	Low
	*A stride iso push in extension from arms and from below creating spinal extensor activation + creating speed peaks and intensity contraction*	4	10x7”	8/10	-	60”	Moderate

**Table 5:** Phase 2A home exercises schedule.

	Home exercises						
	Exercise	Series	Repetitions	Intensity	Duration	Break	Effort
**Block 1**	*Clamshell with blocked lumbar*	3	20–25	8/10	-	-	Moderate
	*Iso push hip extension at 30°*, *standing with blocked core*	3	5x7”	9/10	-	-	High
	*Step with pre-iso push to 3” step*	3	6–8	7/10	-	120”	Low
**Block 2**	*Monsters walk for medial gluteus guided in parallel*	3	15/side	8/10	-	-	Moderate
	*Standing Iso hold anti extensor*	3	8x5”	7/10	-	30”	Low
	*Standing press pallof horizontal over asymmetric ground + bracing*	3	12–15	8/10	1,3,3	-	Moderate

Exercises for this phase are shown in Tables 3–5.

*Phase 2B Posture/alignment*

Phase 2B will comprise between day 38 and day 60 after the intervention. It will follow the same objectives and attributes as phase 2, with the additional instruction of voluntary spine traction movement to enhance posture control. All other aspects will remain unchanged.

*Phase 2C Posture/alignment*

Phase 2C encompassed the period from days 61 to 90 following the intervention. During this phase, the range of motion was increased from 90 degrees of flexion to 85 degrees of extension. To ensure proper exercise adherence, all exercises were performed isometrically. The progression of exercises was customized based on each patient’s ability and level of pain. Exercises were performed using series 1 to 3, with 8 to 15 repetitions, and isometric contractions lasting 5 to 10 seconds. There were 30-second breaks between series, and 2 to 3 minutes between exercises. The exercises performed during this phase are listed in Tables 6–8.

**Table 6:** Phase 2C exercises schedule for the first, third, fifth and seventh sessions.

	Sessions 1, 3, 5 and 7						
	Exercise	Series	Repetitions	Intensity	Duration	Break	Effort
**Block 1**	*Bracing + press pallof vertical a stride*	4	8–10	5/10	1,3,2	-	Low
	*Standing iso hold anti extensor*	3	8x5”	7/10	-	30”	Low
	*Standing hip extension iso push at 30° hip flexion with blocked core*	3	5x7”	9/10	-	-	High
**Block 2**	*Hip extension iso push standing in one leg in asymmetric ground + soft hinge with KB in goblet quad*	3	10	7/10	-	120”	Low
	*A stride ascending row for dorsal extension without blocked lumbar*	3	12–15	8/10	1,3,3	-	Moderate
	*Horizontal press pallof from knees*	3	12	7/10	1,3,2	-	Low
**Block 3**	*One hand from knees with the contrary foot raised*	3	8	8/10	1,2,3	-	Moderate
	*Hip extension in prone position*	3	20–25	8/10	1,3,3	-	Moderate
	*Isotonic clamshell*	3	20–25	8/10	-	120”	Moderate

**Table 7:** Phase 2C exercises schedule for the second, forth, sixth and eight sessions.

	Sessions 2, 4, 6 and 8						
	Exercises	Series	Repetitions	Intensity	Duration	Break	Effort
**Block 1**	*Iso switch dead bug changing support + iso hold anti extension arms + bracing*	3	10	8/10	-	30”	Moderate
	*A stride iso push in extension from arms and from below activating spinal extensors + creating speed peaks and contraction intensity*	4	10x7”	8/10	-	60”	Moderate
	*Bird dog*	3	10	5/10	-	-	Minimal
**Block 2**	*Iso push hip extension standing in one leg over asymmetric stand + iso switch KB changing hands*	3	15	8/10	-	-	Moderate
	*Dorsal Jefferson curl for dorsal with blocked lumbar*	3	8	6/10	-	-	Minimal
	*Post-isometric Koala*	3	10	6/10	-	120”	Minimal
**Block 3**	*Iso holds pelvic retroversion with elastic band standing in asymmetric stand + thoracic flexo-extension with KB in goblet squat*	3	15	7/10	-	-	Low
	*Monsters walk for medial gluteus guided in parallel*	3	15/side	8/10	-	-	Moderate
	*Iso bird dog + row + hip extension in a bench*	3	8/side	8/10	-	120”	Moderate

**Table 8:** Phase 2C exercise schedule for the home exercises.

	Home exercises						
	Exercise	Series	Repetitions	Intensity	Duration	Break	Effort
**Block 1**	*Clamshell with blocked lumbar*	3	20–25	8/10	-	-	Moderate
	*Standing iso push hip extension at 30° with blocked core*	3	5x7”	9/10	-	-	High
	*Step up with pre-iso push to 3” step*	3	6–8	7/10	-	120”	Low
**Block 2**	*Monsters walk for medial gluteus guided in parallel*	3	15/side	8/10	-	-	Moderate
	*Standing anti extensor iso hold*	3	8x5”	7/10	-	30”	Low
	*Standing horizontal press pallof in asymmetric stand + bracing*	3	12–15	8/10	1,3,3	-	Moderate

*Phase 3. Movement strategies*

Phase Three comprises day 90 onward. As per the existing guidelines at this time, the electrode ought to be firmly in place, with minimal probability of relocation. During this phase, the spinal flexion shall be 155 degrees, and the spinal extension shall be 115 degrees. The range of spinal movement shall be complete, enabling patients to incorporate movement into their daily routines. Concentric and eccentric contractions are likewise employed in everyday life. The exercises are listed in Tables 9–12.

**Table 9:** Phase 3 exercise schedule for the first, third and fifth sessions.

	Sessions 1,3 and 5						
	Exercise	Series	Repetitions	Intensity	Duration	Break	Effort
**Block 1**	*Horizontal press pallof from knight with soft core flexion*	3	5 x 3”	8-9/10	-	30”	Moderate
	*Standing iso push hip extension at 30° with blocked core*	3	5x7”	9/10	-	-	High
	*Iso push gluteal bridge + iso hold arms anti extensor + cervical- thoracic flexion facing up + hollowing*	3	5 x 3”	8-9/10	-	30”	Moderate
**Block 2**	*Iso push hip extension standing on leg over asymmetric stand + complete hinge and KB with briefcase hold*	3	10	7/10	2,2,1	120”	Low
	*Iso hold vertical press pallof + switching from symmetric to asymmetric stand*	3	5/side	6/10	-	-	Low
	*Thrust with block and KB in open side*	3	8/side	8/10	-	120”	Moderate
**Block 3**	*Dead weight from block + hip extension with elastic band + bracing*	3	8–12	8/10	1,1,3	-	Moderate
	*Iso push wall drill*	3	15/side	8/10	-	-	Moderate
	*Step hip thrust + bracing*	3	8–12	8/10	1,3,3	120”	Moderate

**Table 10:** Phase 3 exercise schedule for the second, fourth and sixth sessions.

	Sessions 1,3 and 5						
	Exercise	Series	Repetitions	Intensity	Duration	Break	Effort
**Block 1**	*Vertical press pallof from knight with soft core flexion*	3	5 x 3”	8-9/10	-	30”	Moderate
	*Iso push hip extension at 90° de flexion*, *standing with blocked core*	3	5x7”	9/10	-	-	High
	*Iso push gluteal bridge+ iso hold arms anti extensor + cervical- thoracic flexion facing up + hollowing*	3	5 x 3”	8-9/10	-	30”	Moderate
**Block 2**	*Hack squat + bracing*	3	10	7/10	2,2,1	120”	Low
	*Iso hold vertical press pallof + switching from symmetric to asymmetric ground*	3	5/side	6/10	-	-	Low
	*Pendlay row in asymmetric ground with KB*	3	10/side	8/10	1,2,3	60”	Moderate
**Block 3**	*Seal row with extended legs and blocked lumbar with a cushion*	3	10/side	8/10	1,2,3	-	Moderate
	*Dynamic dorsolumbar rotation from thrust*	3	15/side	8/10	-	-	Moderate
	*Lead carries for pelvic stability*	3	10 steps/side	6/10	-	120”	Low

**Table 11:** Phase 3 exercise schedule home exercises A.

	Home exercises A						
	Exercise	Series	Repetitions	Intensity	Duration	Break	Effort
**Block 1**	*Clamshell with blocked lumbar*	3	20–25	8/10	-	-	Moderate
	*Ascending row with dorsal rotation over*	3	5x7”	9/10	-	-	High
	*Post isometric koala*	3	6–8	7/10	-	120”	Low
**Block 2**	*Monster walk for medial gluteus guided in parallel*	3	15/side	8/10	-	-	Moderate
	*Iso push hip internal rotation and flexion from dead bug + forced exhalation*	4	5x5”	9/10	-	-	High
	*Iso push hip extension standing in one leg over asymmetric ground + complete hinge and KB with briefcase hold*	3	10	7/10	2,2,1	120”	Low

**Table 12:** Phase 3 exercise schedule for home exercises B.

	Home exercises B						
	Exercise	Series	Repetitions	Intensity	Duration	Break	Effort
**Block 1**	*Prone lumbar extension*	3	20–25	8/10	-	-	Moderate
	*Jefferson curl without weight*	3	5x7”	9/10	-	-	High
	*Post isometric koala*	3	6–8	7/10	-	120”	Low
**Block 2**	*Dorsolumbar inclination with elastic band*	3	15/side	8/10	-	-	Moderate
	*Iso push internal rotation and hip flexion from dead bug + force exhalation*	4	5x5”	9/10	-	-	High
	*Hip abduction sitting on the floor with fixed back*	3	10	7/10	2,2,1	120”	Low

#### 4.1.2 Spinal cord stimulation proceeding

Spinal cord stimulation (SCS) is a therapy that involves the use of an implantable pulse generator with the potential for enhanced therapeutic success through stimulation algorithms and parameters [28]. By targeting distal areas, such as the dorsal root ganglion, SCS may offer greater anatomical specificity for therapy. Additionally, subthreshold stimulation, utilizing high-frequency or burst energy delivery, has the potential to eliminate noxious and off-target paresthesia. Studies have demonstrated that subthreshold stimulation at high frequencies and/or utilizing different stimulation paradigms can provide equal or even superior pain relief compared with standard SCS [29]. The procedure involves the placement of two octopolar electrodes inserted through the epidural space, positioned beneath the dorsal area posterior to the posterior horn of the spinal cord. The selected level is between the T8 and T11 vertebrae, where the greatest synaptic activity of the spinothalamic tracts responsible for collecting painful sensitivity in the legs and lumbar region is concentrated.

## 5. Patient-reported outcome measures

### 5.1 Primary outcomes

#### 5.1.1 Disability. Oswestry disability index

The Oswestry disability index (ODI) is the most widely used and validated assessment test for lumbar pain. It is a self-assessment test divided into ten sections designed to assess limitations in daily life. Each section has a score ranging from 0 to 5, where 5 was the highest instability level, with the maximum possible score being 50 points [39]. The total rate will be calculated by dividing the participant’s score by the maximum possible score and dividing it by 100 to obtain a percentage [40]. A higher score on the questionnaire indicates greater disability associated with low back pain [41]. ODI is widely regarded as the “gold standard” among tools for assessing functional outcomes in low back conditions [42]. The ODI index is validated to Spanish [43] presenting high sensitivity and specificity to evaluate the function [44,45].

### 5.2 Secondary outcomes

#### 5.2.1 Quality of life. 36-Item short form health survey

The *36-Item Short Form Health Survey (*SF-36) measures quality of life and comprises several dimensions: (a) physical functioning, (b) role physical, (c) role emotional, (d) social functioning, (e) bodily pain, and (f) vitality, as well as two general dimensions that encompass all the dimensions: a) general health and (b) mental health. The first four subtitles assess the physical health, while the last four assess the mental health [46]. The scale is evaluated between 0 to 100 and high score indicates a better health level, while a low score indicates poor quality of life. It is a widely used instrument in healthcare that highlights the satisfactory psychometric properties of internal consistency and test-retest reliability [47].

#### 5.2.2 Pain perception. Visual analogue scale

The visual analogue scale (VAS, values from 0 to 10) will be used to assess the subjective perception pain (0 will be considered to reflect non-existence of pain and 10 as the worst/intolerable pain). Research personnel will score the paper-based VAS using a ruler to measure the distance (cm) from the left end of the VAS scale to the patients’ marks, obtaining the average VAS value [48]. Previous studies have shown that the VAS scale demonstrated high reliability coefficients (α = 0.98) [49].

### 5.3 Exploratory analysis

#### 5.3.1 Patient’s satisfaction

Patient satisfaction involves the subjective evaluation of treatment effectiveness, health services, and healthcare providers. It represents a complex construct that should not be simplified into a single, one-dimensional item. The Pain Treatment Satisfaction Scale (PTSS) was created to evaluate satisfaction levels among patients experiencing both acute and chronic pain [50]. There are already published studies about diagnosed patients that evaluate their satisfaction using a numeric scale of 11 (-5 to 5) [51,52]. High scores indicate patient satisfaction with the treatment.

#### 5.3.2 Fear of movement. Tampa scale of Kinesiophobia

The TSK will be used to measure fear of movement or reinjury. The TSK is a self-administered questionnaire composed of different questions with a 4-point Likert scale ranging from “strongly disagree” to “strongly agree.” Higher scores indicate greater fear of movement or re-injury, whereas lower scores indicate less fear. The internal consistency of the TSK scores ranged from α = 0.90 with high test-retest reliability {ICC (2,1) = 0.934}[53]. TSK values will be collected following the same data collection protocol.

#### 5.3.3 Self-efficacy

Self-efficacy is a powerful predictor of motivation and learning in people [54]. The self-efficacy questionnaire is composed of 19 items with 3 domains that assess self-efficacy for pain management and physical functioning [55]. These domains analyze limitations in work, social activities, and self-care activities [56]. The Graded Chronic Pain Scale (GCPS) employs 19 questions to identify the chronicity of pain and its impact on the patient [57]. Patients affected by chronic pain may experience it at different sites. This scale addresses pain in general, without classifying it separately [58]. The Spanish version of the Graded Chronic Pain Scale had a high internal consistency (α = 0.87) [59].

#### 5.3.4 Pain catastrophizing scale

The Pain Catastrophizing Scale (PCS), a self-administered questionnaire (13 items on a Likert-type scale from 0 to 4), will be used in this study to assess the level of catastrophizing in the presence of pain [60]. The dimensionality of the scale is characterized by three interrelated factors, which are helplessness, rumination, and magnification, describing a single second-order latent construct (catastrophizing) [61]. The total score ranges from 0 to 52 points, with higher scores representing higher levels of catastrophizing. Studies have reported good levels of content and construct validity, internal consistency, and test-retest reliability for the PCS in examining various musculoskeletal disorders [62,63] and different language versions [64]. In particular, the Spanish version of the PCS has an internal consistency of 0.79 and a test-retest reliability of 0.84 [65]. Low scores indicate low levels of catastrophism and high values show high levels of catastrophism [65,66].

## 6. Program feasibility and safety: Attendance and compliance

There are numerous obstacles and factors that affect the adherence to exercise for individuals with chronic lower back pain [67]. Patients with spine pain are increasingly seeking personalized exercise programs [68]. However, studies have shown that home-based interventions supported by individualized video-based exercise programs result in greater improvement following treatment [69]. The proposed intervention in this study combines these two perspectives. During the initial phases, individualized training will be provided to ensure compliance with the treatment plan and minimize potential complications. Additionally, once the patient begins working from home, the exercises will be accompanied by videos to facilitate daily monitoring of the intervention. Moreover, WHO defines adherence as “the extent to which a person’s behaviour—taking medication, following a diet, and/or executing lifestyle changes—corresponds with the agreed recommendations from a healthcare provider". In the therapeutic exercise group, participants will be encouraged to follow the guidelines at home to complement the intervention. The exercise group should perform the therapeutic exercises according to the instructed regularity as per the SIRAS scale [70].

## 7. Oversight and monitoring

The investigator members (J.V-M and F.S.M) who concurred on the study design and applied for funding will comprise the trial steering committee. The principal responsibility of both principal investigators will be to coordinate the execution of the study within the Pain Unit. The remaining participants will actively contribute to the coordination of the patients, application of the treatments, and management of any adverse effects that may arise. In the event of any serious adverse event occurring during the study, the principal investigators will notify the research ethics committee of the “Comité de Ética de la Investigación con medicamentos del Área de Salud de Salamanca” (IBSAL) to take the necessary measures. Furthermore, patients will be contacted by telephone during and after the trial to monitor for any adverse effects.

## 8. Data collection and analysis

### 8.1 Data collection

The medical records of patients will be inputted directly into the computer system, which is securely stored in the facilities where the evaluation will take place. To facilitate the exchange of data for further analysis among researchers, an Excel spreadsheet will be sent, containing only the unique identification number of each patient. This approach ensures the confidentiality and security of the data.

### 8.2 Statistical analysis

#### 8.2.1 Baseline characteristics

To check whether outcome and demographic baseline measures will be balanced among intervention groups, comparisons will be conducted using analyses of variance (ANOVA) or chi-square tests (i.e., SCS group and EX+SCS group) to analyze significant differences between groups (p>0.05)

#### 8.2.2 Analysis of the outcome measures

Following the Consolidated Standards of Reporting Trials (CONSORT) guidelines on the reporting of RCTs, a per-protocol analysis will be performed. The normality assumption will be checked with the Kolmogorov–Smirnov test and box-plot analysis, while the homogeneity of the variance will be tested through the Levene test. To analyze the acute and short-term effects of SCS and SCS combined with core and control motor exercise on PSPS T2, a two-way ANOVA for repeated measures with experimental groups (i.e., Post3weeks, Post2months, Post6months) as factors will be used, followed by Tukey corrections, to examine time, group, and interaction effects through within- and between-group comparisons (primary and secondary outcomes). The results will be presented as the mean difference (MD) and confidence interval at 95% (IC95%). Effect size (ES) will be estimated by calculating Cohen’s d coefficient. All analyses will be performed using the statistical analysis software SPSS 24 (IBM Inc., Chicago, Illinois, USA). In the case of dropouts during the study or if the statistical power is less than the established 80%, an intention-to-treat principle analysis will be performed [71]. The normality of the data will be tested by visual inspection of histograms, and the characteristics of the participants will be presented using descriptive statistical tests. To assess between-group differences in response to treatment at each post-baseline time point, the mean between-group differences and their associated 95% confidence intervals (CI) will be calculated by constructing mixed linear models using interaction terms (group vs. time). The treatment effects will be adjusted by including baseline outcome values as covariates in the model. Statistical analysis will be conducted by a researcher who is not involved in any of the phases of data collection and will receive data in coded form. Minimal clinically important differences are patient-derived scores that reflect changes in a clinical intervention that are meaningful to the patient. To do this from the delta percentage (Δ%) will calculate using the standard formula: change (%) = [(post-test score − pre-test score)/pre-test score] × 100.

#### 8.2.3 Correlation coefficient

The strength of the relationship between the variables will be examined using the Pearson correlation coefficient and/or Spearman correlation coefficient (for non-compliance with the normality assumption).

## 9. Dissemination plan

The dissemination of research findings is an essential component of the research process, and this study places considerable emphasis on various aspects of it. The results of this study will be published in scientific journals that are focused on the fields of medicine, physiotherapy, and exercise. By being published, the study will be shared with the broader scientific community, ensuring that the knowledge gained from the research is accessible to professionals and researchers in these fields. Moreover, all members of the research team are highly skilled in the areas of exercise and spinal cord stimulation, and they are also experienced researchers in their respective fields. This allows them to make valuable contributions to the development of articles during the future publication process. Additionally, the protocols implemented for the treatments will be described in detail to enhance transparency and increase the reproducibility of the study’s results. It is important to note that the study’s results may be provided to third parties if there is a justifiable cause and it is accepted by the authors.

## Supporting information

S1 File (PDF)

S2 File (PDF)

## Abbreviations

FBSS: Failed back surgery syndrome
PSPS-T2: Persistent Spinal Pain Syndrome
SCS: Spinal cord stimulation
RCT: Randomized controlled trial
VAS: Visual Analogue Scale
TSK: Tampa Scale of Kinesiophobia
ODI: Oswestry Disability Index
