# Supplementary material for: Spinal cord stimulation combined with exercise in patients diagnosed with persistent spinal pain syndrome. Study protocol for a randomized control trial
Source: PLoS One. 2024 Oct 31;19(10):e0309935. doi: 10.1371/journal.pone.0309935 (PMC11527166; doi:10.1371/journal.pone.0309935)
Supplement: S1 File — (PDF) [file pone.0309935.s001.pdf]

ClinicalTrials.gov Protocol Registration and Results System (PRS) Receipt
Release Date: February 23, 2024

ClinicalTrials.gov ID: NCT06272539

Study Identification

Unique Protocol ID: 2023 101435

Brief Title: Spinal Cord Stimulation Combined With Exercise in Persistent Spinal Pain Syndrome

Official Title: Spinal Cord Stimulation Combined With Exercise in Patients Diagnosed With Persistent Spinal Pain Syndrome. A Randomized Control Trial (RCT)

Secondary IDs:

Study Status

Record Verification: February 2024

Overall Status: Not yet recruiting

Study Start: April 1, 2024 [Anticipated]

Primary Completion: January 1, 2026 [Anticipated]

Study Completion: January 1, 2026 [Anticipated]

Sponsor/Collaborators

Sponsor: Fundación Universidad Católica de Valencia San Vicente Mártir

Responsible Party: Sponsor

Collaborators: Instituto de Investigación Biomédica de Salamanca

Oversight

U.S. FDA-regulated Drug: No

U.S. FDA-regulated Device: No

U.S. FDA IND/IDE: No

Human Subjects Review: Board Status: Approved
Approval Number: ID: 2023 10 1435
Board Name: CONCEPCIÓN TURRIÓN GÓMEZ
Board Affiliation: Comité de Ética de la Investigación con medicamentos del Área de Salud de Salamanca
Phone: 923291100
Email: comite.etico.husa@saludcastillayleon.es
Address:

Paseo de San Vicente, 58-182
37007 Salamanca

Data Monitoring: No

FDA Regulated Intervention: No

Study Description

Brief Summary: Introduction. At the neurophysiological level, it is possible to observe an increase in the central processing of pain in patients diagnosed with persistent Spinal Pain Syndrome (PSPS-T1/2), potentially stemming from dysfunctions in the endogenous facilitation and inhibition of pain. Administration of high doses of spinal cord stimulation to individuals with PSPS-T1/2 may induce supraspinal descending activation. Similarly, exercise is recognized as a fundamental aspect of spinal pain management. Studies have demonstrated its impact on neurophysiological factors, including the release of spinal and supraspinal beta-endorphins, which activate μ-opioid receptors. Therefore, the purpose of this study will be to examine the effect of SCS in combination with lumbo-pelvic stability core training on perceived low back pain, quality of life and disability in failed back surgery syndrome (FBSS) patients. Methods/ Materials. A double-blind randomized clinical trial (RCT) has been designed. All participants will be randomized from a pre-set sequence. The intervention design has been elaborated from the CONSORT guidelines. This study protocol has been approved by the Ethics Committee in research of Salamanca Health Area (protocol number PI 2023 101435 in (24/01/2024) in accordance with the ethical guidelines of the Helsinki declaration. Sample size was calculated using G Power® Sample size software (University of Düsseldorf). The calculation was based on a moderate effect size of 0.4 (partial $\eta 2 = 0.40$, $\alpha = .05$, power = 0.90), resulting in a total of 28 patients. Assuming a 30% dropout rate, 36 participants will be recruited in total. Two sessions per week will be scheduled for 8 weeks with a total of 16 sessions. Each work session will have a duration of 60 minutes. The exercise will be adapted according to the phases based on the results already published, limiting in each phase the degrees of flexion and extension of the spine in order to avoid the risk of electrode migration. Primary outcomes will be functionality, satisfaction, strength, psychosocial variables, quality of life and pain perception.

Detailed Description:

Conditions

Conditions: Failed Back Surgery Syndrome

Keywords: Spinal Cord Stimulation
Exercise

Study Design

Study Type: Interventional

Primary Purpose: Treatment

Study Phase: N/A

Interventional Study Model: Parallel Assignment

Number of Arms: 2

Masking: Triple (Care Provider, Investigator, Outcomes Assessor)
By doing so, we will aim to eliminate or reduce potential biases due to the order of patients and any transference effects that might occur if one profile clinical patients will influence the performance in several evaluations. Specifically, each

	patient will complete a sequence of allocation in the order of A, B, B, A, where 'A' represents SCS treatment isolated and 'B' denotes combined treatment. Subsequently, the average of the two 'A' conditions was calculated, and the same process was applied for the 'B' conditions
Allocation:	Randomized
Enrollment:	36 [Anticipated]

Arms and Interventions

Arms	Assigned Interventions
Active Comparator: Spinal Cord Stimulation<br>Spinal cord stimulation (SCS) involves an implantable pulse generator with the potential for enhanced therapeutic success through stimulation algorithms and parameters (28). Spinal cord stimulation (SCS) targeting distal areas, such as the dorsal root ganglion, may offer greater anatomical specificity in therapy. Subthreshold stimulation, utilizing high-frequency or burst energy delivery, has the potential to eliminate noxious and off-target paresthesiae. Recent studies have demonstrated that subthreshold stimulation at high frequencies and/or utilizing different stimulation paradigms can provide equal or even superior pain relief compared to standard SCS (29). The procedure entails the placement of two octapolar electrodes inserted through the epidural space, positioned beneath the dorsal area posterior to the spinal cord's posterior horn.	Procedure/Surgery: Spinal Cord Stimulation<br>Spinal cord stimulation (SCS) involves an implantable pulse generator with the potential for enhanced therapeutic success through stimulation algorithms and parameters (28). Spinal cord stimulation (SCS) targeting distal areas, such as the dorsal root ganglion, may offer greater anatomical specificity in therapy
Experimental: Spinal Cord Stimulation+Exercise<br>The experimental group will perform a Lumbo-pelvic core stability training program combined with motor control exercises through specific therapeutic exercises of the lumbopelvic centre combined with neurostimulation treatment. The exercise will be adapted according to the phases based on the results already published, the following intervention plan has been designed. Additionally, in each of the phases, the exercises were designed, limiting the degree of flexion/extension and lumbar traction of the exercises. Two weekly sessions will be scheduled during 8 weeks with a total of 24 sessions, each one of 60 minutes of duration. A certified physiotherapist in exercised with at least 10 years of clinical practice has applied treatment.	Procedure/Surgery: Exercise<br>The exercise The exercise will be adapted according to the phases based on the results already published, limiting in each phase the degrees of flexion and extension of the spine in order to avoid the risk of electrode migration.<br>Procedure/Surgery: Spinal Cord Stimulation<br>Spinal cord stimulation (SCS) involves an implantable pulse generator with the potential for enhanced therapeutic success through stimulation algorithms and parameters (28). Spinal cord stimulation (SCS) targeting distal areas, such as the dorsal root ganglion, may offer greater anatomical specificity in therapy

Outcome Measures

Primary Outcome Measure:

1. Disability (Oswestry Disability Index)
   The Oswestry Disability Index (ODI) is the most used and validated assessment test for lumbar pain. Is a self-assessment test divided in ten sections designed to assess the limitations in daily life

   [Time Frame: at baseline, Post3weeks, Post2months, Post6months]
2. Perception Pain (Visual analogue scale)
   This scale is an efficient tool to quantify in a subjective and selective way this range in which 0 is considered a total absence of pain and 10 the worst pain imaginable

[Time Frame: at baseline, Post3weeks, Post2months, Post6months]

Secondary Outcome Measure:

3. Quality of life (Short Form 36 Health Survey, SF36)
   The measures the quality of life and comprises several dimensions: (a) physical functioning, (b) role physical, (c) role emotional, (d) social functioning, (e) bodily pain and (f) vitality. Scores for each component summary are calculated based on responses to the twelve items, with higher scores indicating better health-related quality of life.

   [Time Frame: at baseline, Post3weeks, Post2months, Post6months]
4. Patient's satisfaction
   There are already published studies about diagnosed patients that evaluate their satisfaction using a numeric scale of 11 (-5 to 5) (37,38). High scores show the patient's satisfaction with the treatment.

   [Time Frame: at baseline, Post3weeks, Post2months, Post6months]
5. Strength (Sorensen Test)
   Sorensen test it measures the amount of strength and resistance of the back extensors

   [Time Frame: at baseline, Post3weeks, Post2months, Post6months]
6. Fear of Movement (Tampa Scale of Kinesiophobia, TSK)
   The Tampa Scale of Kinesiophobia (TSK) was used to measure fear of movement or reinjury. Scores on the TSK are calculated by summing responses to individual items, with higher scores indicating greater levels of kinesiophobia. The scale has been validated and widely used in research and clinical settings to assess and monitor fear of movement in individuals with chronic pain conditions, musculoskeletal injuries, and other conditions where fear of movement may impact rehabilitation or daily functioning.

   [Time Frame: at baseline, Post3weeks, Post2months, Post6months]
7. Individual's belief in their own ability to successfully execute tasks (Self-efficacy Scale)
   The Self-Efficacy Scale is a tool used to measure an individual's belief in their own ability to successfully execute tasks and achieve goals in specific situations.

   The scale typically consists of items that assess an individual's perceived self-efficacy in various domains or activities, such as academic performance, athletic ability, social interactions, or coping with challenges. Respondents rate their confidence levels on a Likert scale, indicating the extent to which they believe they can successfully perform tasks or overcome obstacles.

   Scores on the Self-Efficacy Scale are calculated by summing responses to individual items, with higher scores indicating greater perceived self-efficacy in the specified domains.

   [Time Frame: at baseline, Post3weeks, Post2months, Post6months]
8. Catastrophic thinking (Pain Catastrophizing Scale)
   The Pain Catastrophizing Scale (PCS), a self-administered questionnaire (13 items on a Likert-type scale from 0 to 4) was used in this study to assess the level of catastrophizing in the presence of pain

   [Time Frame: at baseline, Post3weeks, Post2months, Post6months]

Eligibility

Minimum Age: 18 Years

Maximum Age: 80 Years

Sex: All

Gender Based: No

Accepts Healthy Volunteers: No

Criteria: Inclusion Criteria:

- Have a diagnostic of PSPS-T1/2 with leg pain and back pain,
- Patients older than 18 years

- 6 months with pain
- Visual Analogue Scale score >7
- Spanish native language

Exclusion Criteria:

- Previous surgeries in abdominal area
- Pregnant or lactating
- Severe fractures or pathologies
- Spine structural deformity
- Neurologic or psychiatric issues.

Contacts/Locations

Central Contact Person: Juan Vicente Mampel, PhD
Telephone: 674177877
Email: juan.vicente@ucv.es

Central Contact Backup:

Study Officials: Francisco J Sanchez Montero
Study Principal Investigator
Complejo Asistencial Universitario de Salamanca. Unidad del Dolor

Juan Vicente-Mampel
Study Principal Investigator
Catholic University of Valencia

Locations: **Spain**
Juan Vicente-Mampel
Torrent, Valencia, Spain, 46900
Contact: Juan V Vicente-Mampel, PhD 674177877 juan.vicente@ucv.es

IPDSharing

Plan to Share IPD: No

References

Citations: Othman R, Dassanayake S, Jayakaran P, Tumilty S, Swain N, Mani R. Relationships Between Psychological, Social, Physical Activity, and Sleep Measures and Somatosensory Function in Individuals With Spinal Pain: A Systematic Review and Meta-analysis. Clin J Pain. 2020 Feb;36(2):124-134. doi: 10.1097/AJP.0000000000000775. PubMed 31764166

Baber Z, Erdek MA. Failed back surgery syndrome: current perspectives. J Pain Res. 2016 Nov 7;9:979-987. doi: 10.2147/JPR.S92776. eCollection 2016. PubMed 27853391

Christelis N, Simpson B, Russo M, Stanton-Hicks M, Barolat G, Thomson S, Schug S, Baron R, Buchser E, Carr DB, Deer TR, Dones I, Eldabe S, Gallagher R, Huygen F, Kloth D, Levy R, North R, Perruchoud C, Petersen E, Rigoard P, Slavin K, Turk D, Wetzel T, Loeser J. Persistent Spinal Pain Syndrome: A Proposal for Failed Back Surgery Syndrome and ICD-11. Pain Med. 2021 Apr 20;22(4):807-818. doi: 10.1093/pm/pnab015. PubMed 33779730

Nijs J, Meeus M, Cagnie B, Roussel NA, Dolphens M, Van Oosterwijck J, Danneels L. A modern neuroscience approach to chronic spinal pain: combining pain neuroscience education with cognition-targeted motor control training. Phys Ther. 2014 May;94(5):730-8. doi: 10.2522/ptj.20130258. Epub 2014 Jan 30. PubMed 24481595

Cho JH, Lee JH, Song KS, Hong JY. Neuropathic Pain after Spinal Surgery. Asian Spine J. agosto de 2017;11(4):642-52. 8. Nie C, Chen K, Chen J, Zhu Y, Jiang J, Jin X, et al. Altered central pain processing assessed by quantitative sensory testing in patients with failed back surgery syndrome

Links:

Available IPD/Information:
