# Supplementary material for: Spinal cord stimulation combined with exercise in patients diagnosed with persistent spinal pain syndrome. Study protocol for a randomized control trial
Source: PLoS One. 2024 Oct 31;19(10):e0309935. doi: 10.1371/journal.pone.0309935 (PMC11527166; doi:10.1371/journal.pone.0309935)
Supplement: S2 File — (PDF) [file pone.0309935.s002.pdf]

STANDARD PROTOCOL ITEMS: RECOMMENDATIONS FOR INTERVENTIONAL TRIALS

SPIRIT 2013 Checklist: Recommended items to address in a clinical trial protocol and related documents*

Section/item	Item No	Description
**Administrative information**		
Title	1	Spinal cord stimulation combined with exercise in patients diagnosed with persistent spinal pain syndrome. Proposal for a randomized control trial (RCT).
Trial registration	2a	Trial identifier (NCT06272539). Spinal cord stimulation combined with exercise in patients diagnosed with persistent spinal pain syndrome. A randomized control trial (RCT).

2b

1. Primary registry and trial-identifying number: Spinal cord neurostimulation combined with exercise in patients diagnosed with persistent spinal pain syndrome. Proposal for a randomized control trial. NCT06272539.
2. Date of registration in primary registry: April 1, 2024
3. Secondary identifying numbers: Ethics Committee in research of the Área de Salud de Salamanca (protocol number PI 2023 101435)
4. Sources of monetary or material support: This research did not receive any specific grant from funding agencies in the public, commercial, or not-for-profit sectors.
5. Primary sponsor: Vicente-Mampel, J
6. Secondary sponsor(s): Blanco-Giménez, P
7. Contact for public queries: paula.blanco@ucv.es ; +34 618475668; Faculty of Medicine and Health Science. Department of Physiotherapy, Catholic University of Valencia, Torrent, Valencia, Spain.
8. Contact for scientific queries: Not applicable.
9. Public title: Spinal cord stimulation combined with exercise in patients diagnosed with persistent spinal pain syndrome.
10. Scientific title: Spinal cord stimulation combined with exercise in patients diagnosed with persistent spinal pain syndrome. Proposal for a randomized control trial (RCT).
11. Countries of recruitment: Salamanca, Spain.
12. Health condition(s) or problem(s) studied: Patients diagnosed with Persistent Spinal Pain Syndrome (PSPS-T1/T2).
13. Intervention(s): All participants will be randomized from a pre-set sequence. Two sessions per week will be scheduled for 8 weeks with a total of 16 sessions. Each work session will have a duration of 60 minutes. The exercise will be adapted according to the phases based on the results already published, limiting in each phase the degrees of flexion and extension of the spine to avoid the risk of electrode migration.
14. Key inclusion and exclusion criteria: The eligibility inclusion criteria were: Have a diagnostic of PSPS-T1/2 with leg pain and back pain, ii) patients older than 18 years, iii) ≥6 months with pain, iv) VAS score >7 and v) Spanish native language. Exclusion criteria were: (i) Previous surgeries in abdominal area; (ii) Pregnant or lactating; (iii) Severe fractures or pathologies; (iv) Spine structural deformity; (vi) Neurologic or psychiatric issues.
15. Study type: This study was designed a double-blinded comparative longitudinal and prospective randomized controlled trial (RCT).
16. Date of first enrolment: April 1, 2024
17. Target sample size: 36 participants will be recruited in total.
18. Recruitment status: Pending: Participants are not yet being recruited or enrolled at any site.

19. Primary outcome(s): - *Oswestry Disability Index:* Each section has score that ranges from 0 to 5, where 5 is the highest instability level.

- *Quality of life (SF-36):* The SF-36 measures the quality of life and comprises several dimensions: (a) physical functioning, (b) role physical, (c) role emotional, (d) social functioning, (e) bodily pain and (f) vitality;
- *Visual analogue scale (VAS):* We will use the scale that provides values from 0 to 10. This scale is an efficient tool to quantify in a subjective and selective way this range in which 0 is considered a total absence of pain and 10 the worst pain imaginable.

20. Key secondary outcome(s): - Patient's satisfaction: There are already published studies about diagnosed patients that evaluate their satisfaction using a numeric scale of 11 (-5 to 5) (37,38). High scores show the patient's satisfaction with the treatment.

- *Sorensen Test: Sorensen test it measures the amount of strength and resistance of the back extensors. The subject is asked to be going prone position against a table and to keep the trunk parallel to the floor against gravity once the legs and pelvis are flexed. The amount of time in this position will be measured in seconds. (39)*
- *Tampa Scale of Kinesiophobia: The TSK is a self-administered questionnaire composed of different questions with a 4-point Likert scale ranging from "strongly disagree" to "strongly agree."*
- *Self-efficacy: Self-efficacy questionnaire is composed of 19 items with 3 domains that assess self-efficacy for pain management and physical functioning.*
- *Pain Catastrophizing Scale: The Pain Catastrophizing Scale (PCS), a self-administered questionnaire (13 items on a Likert-type scale from 0 to 4) was used in this study to assess the level of catastrophizing in the presence of pain. The total scores range from 0 to 52 points, where higher scores represent higher levels of catastrophizing.*

Protocol version	3	Date and version identifier<br>- Study Start: April 1, 2024<br>- Study Completion: January 1, 2026<br>- Authors: JVM, FFV, DSP, FHZ, MMS, PBG, FJSM
Funding	4	This research did not receive any specific grant from funding agencies in the public, commercial, or not-for-profit sectors.
Roles and responsibilities	5a	All the authors were involved in the conception, design, and methodology of the protocol. JVM drafted the manuscript. FSM revised the manuscript for critically intellectual content. PBG and FFV reviewed and edited the manuscript. All authors read and approved the final manuscript for submission.

5b School of Medicine and Health Science. Department of Physiotherapy, Catholic University of Valencia, Torrent, Valencia, Spain **and** Anesthesiology Service, Pain Unit. Complejo Asistencial Universitario de Salamanca, Salamanca, Spain (CAUSA).

5c The centers to which the researchers are affiliated did not intervene in the design of this study and will not have any role during its execution, analysis, and interpretation of the data, nor in the decision to present the results.

5d "Study Oversight Committee. Composition of the Research Ethics Committee (CEIm) of the Salamanca Health Area President: Head of the General Surgery and Digestive System Service; Vice President: Data Protection Officer of CAUSA; Secretary: Pharmacist and Biochemist; Scientific Committee Representative.
IBSAL Members: Clinical Pharmacologist ASCOL President (patient representative) and Clinical Trials Manager.

Background and rationale	6a	**Background.** At the neurophysiological level, it is possible to observe an increase in the central processing of pain in patients diagnosed with PSPS-T1/2, potentially stemming from dysfunctions in the endogenous facilitation and inhibition of pain. Administration of high doses of spinal cord stimulation to individuals with PSPS-T1/2 may induce supraspinal descending activation. Similarly, exercise is recognized as a fundamental aspect of spinal pain management. Studies have demonstrated its impact on neurophysiological factors, including the release of spinal and supraspinal beta-endorphins, which activate μ-opioid receptors. Therefore, the purpose of this study will be to examine the effect of SCS in combination with lumbo-pelvic stability core training on perceived low back pain, quality of life and disability in FBSS patients.<br><br>***Existing knowledge related to neurostimulation in FBSS:*** *S*pinal cord neurostimulation has a medium-term effect (up to 2 or 3 years), with a grade of recommendation B (13). At the neurophysiological level, an increase in the central processing of pain in patients with failed back surgery can be established, which may be caused by a dysfunction of the endogenous facilitation and inhibition of pain. In fact, spinal cord stimulation produces effects on the descending modulatory centres of pain also produced by electrical stimulation of the spinal cord, considering that high doses of spinal stimulation in humans diagnosed with failed back surgery could generate supraspinal descending inhibitory modulatory effects capable of generating analgesia (14).<br><br>***Existing knowledge related to exercise in FBSS:*** Conservative intervention, led by exercise, is considered to be the cornerstone of spinal pain treatment (17). Looking at the results from spinal pain labels other than FBSS shows that exercises focused on motor control and spinal stabilization have been shown to be more effective than surgical and pharmacological interventions in reducing pain and decreasing disability (19,20).
	6b	This underscores the imperative for a multidimensional approach. Treatment should concentrate on enhancing the patient's perceptions and beliefs about movement, addressing not only mechanical and functional parameters but also improving the overall experience. (23). This means that there are higher efferent nociceptive processes that can explain the worsening of the symptomatology without the mechanical trigger being the possible cause of this pain. The use of both treatment techniques (stimulation/exercise) results in average evidence, presenting low effect measurements if applied in isolation (24).

Objectives	7	To evaluate the effect of combined treatment of spinal neurostimulation and exercise focused on neuromuscular control of the CORE in comparison with isolated treatment of spinal stimulation on functionality, pain, psychosocial variables, patient satisfaction and quality of life in patients diagnosed with PSPS-T1/2 in combination with spinal cord stimulation. The hypothesis describe a combined intervention (SCS + EXE) is better than an existing standard isolated treatment (SCS). The null hypothesis in this case states that there is no difference between the treatments.
Trial design	8	This study was designed a double-blinded comparative longitudinal and prospective randomized controlled trial (RCT). Once the groups have been established there will we four sample collections: Pre, $Post_{3weeks}$, $Post_{2months}$, $Post_{6months}$. After taking sample Pre and before the surgery, patients will be introduced to the CORE neuromuscular and control motor exercises protocol.

Methods: Participants, interventions, and outcomes

Study setting	9	The study will be performed at the Complejo Asistencial Universitario de Salamanca, Spain (CAUSA). On a sample of patients diagnosed with diagnosed with PSPS-T1/2 on the waiting list to receive a spinal neurostimulation device at the Pain Unit of the Salamanca Hospital.
Eligibility criteria	10	The eligibility inclusion criteria were: Have a diagnostic of PSPS-T1/2 with leg pain and back pain, ii) patients older than 18 years, iii) ≥6 months with pain, iv) VAS score >7 and v) Spanish native language. Exclusion criteria were: (i) Previous surgeries in abdominal area; (ii) Pregnant or lactating; (iii) Severe fractures or pathologies; (iv) Spine structural deformity; (vi) Neurologic or psychiatric issues.

Interventions	11a	**Active Comparator:** Spinal Cord Stimulation Spinal cord stimulation (SCS) involves an implantable pulse generator with the potential for enhanced therapeutic success through stimulation algorithms and parameters. Spinal cord stimulation (SCS) targeting distal areas, such as the dorsal root ganglion, may offer greater anatomical specificity in therapy. Subthreshold stimulation, utilizing high□frequency or burst energy delivery, has the potential to eliminate noxious and off-target paresthesiae. Recent studies have demonstrated that subthreshold stimulation at high frequencies and/or utilizing different stimulation paradigms can provide equal or even superior pain relief compared to standard SCS. The procedure entails the placement of two octopolar electrodes inserted through the epidural space, positioned beneath the dorsal area posterior to the spinal cord's posterior horn. **Experimental: Spinal Cord Stimulation+Exercise** The experimental group will perform a Lumbo-pelvic core stability training program combined with motor control exercises through specific therapeutic exercises of the lumbopelvic centre combined with neurostimulation treatment. Based on the principles established by Falla et al., the following intervention plan has been designed. Additionally, in each of the phases, the exercises were designed, limiting the degree of flexion/extension and lumbar traction of the exercises. Two weekly sessions will be scheduled during 8 weeks with a total of 24 sessions, each one of 60 minutes of duration. A certified physiotherapist in exercised with at least 10 years of clinical practice has applied treatment.
	11b	If pain was experienced during the application of the exercise protocol, adjustments would be made to the parameters of range of motion, joint position, and exercise intensity in an effort to minimize changes to the stimulation of the target tissue
	11c	Strategies to improve adherence to intervention protocols will be exercise group should perform the therapeutic exercises following the instructed regularity according to the SIRAS scale.
	11d	It will be prohibited to perform exercise other than that established during the duration of the research. Additionally, patients will have limited the type of activities as currently presented within the established protocol.

Outcomes	12	Primary Outcome Measure: 1. Oswestry Disability Index The ODI is the most used and validated assessment test for lumbar pain. Is a self-assessment test divided in ten sections designed to assess the limitations in daily life [Time Frame: at baseline, Post3weeks, Post2months, Post6months] 2. Visual analogue scale. This scale is an efficient tool to quantify in a subjective and selective way this range in which 0 is considered a total absence of pain and 10 the worst pain imaginable [Time Frame: at baseline, Post3weeks, Post2months, Post6months. Secondary Outcome Measure: 3. Quality of life The measures the quality of life and comprises several dimensions: (a) physical functioning, (b) role physical, (c) role emotional, (d) social functioning, (e) bodily pain and (f) vitality [Time Frame: at baseline, Post3weeks, Post2months, Post6months] 4. Patient's satisfaction There are already published studies about diagnosed patients that evaluate their satisfaction using a numeric scale of 11 (-5 to 5) (37,38). High scores show the patient's satisfaction with the treatment. [Time Frame: at baseline, Post3weeks, Post2months, Post6months] 5. Sorensen Test Sorensen test it measures the amount of strength and resistance of the back extensors [Time Frame: at baseline, Post3weeks, Post2months, Post6months] 6. Tampa Scale of Kinesiophobia The Tampa Scale of Kinesiophobia (TSK) was used to measure fear of movement or reinjury. [Time Frame: at baseline, Post3weeks, Post2months, Post6months] 7. Self-efficacy Self-efficacy questionnaire is composed of 19 items with 3 domains that assess self-efficacy for pain management and physical functioning [Time Frame: at baseline, Post3weeks, Post2months, Post6months] 8. Pain Catastrophizing Scale The Pain Catastrophizing Scale (PCS), a self-administered questionnaire (13 items on a Likert-type scale from 0 to 4) was used in this study to assess the level of catastrophiz-ing in the presence of pain [Time Frame: at baseline, Post3weeks, Post2months, Post6months]
Participant timeline	13	There will be realized two weekly sessions that will be scheduled during 8 weeks with a total of 24 sessions, each one of 60 minutes of duration. Once the groups will be establish there will we four sample collections: Pre, $Post_{3weeks}$, $Post_{2months}$, $Post_{6months}$.

Sample size	14	Due to the absence of similar studies that allow the calculation of the sample size on the basis of an unknown effect size, an intervention design with a preliminary sample size of 36 subjects (18 participants per group) was developed. The sample size was calculated using the G Power® Sample size software (University of Düsseldorf). The calculation was based on a moderate effect size of 0.4 (partial η2 = 0.40, α = .05, power = 0.90) a total of 28 patients in total were calculated. Assumed 30% losses will recruit 36 participants in total at the end. The possibility of increasing the number of subjects will be considered in case of low statistical power to reach these predefined levels. In case of dropouts, non-compliance or absence of results, an intention-to-treat analysis will be performed.
Recruitment	15	The patients will be those who are on the waiting list to receive a spinal neurostimulator in the in the Pain Unit of the Salamanca Hospital, and who meet the requirements of the study. Recruitment will be made directly from the waiting lists for this surgical intervention.

Methods: Assignment of interventions (for controlled trials)

Allocation:

Sequence generation	16a	By doing so, we will aim to eliminate or reduce potential biases due to the order of patients and any transference effects that might occur if one profile clinical patients will influence the performance in several evaluations. Specifically, each patient will complete a sequence of allocation in the order of A, B, B, A, where 'A' represents SCS treatment isolated and 'B' denotes combined treatment. Subsequently, the average of the two 'A' conditions was calculated, and the same process was applied for the 'B' conditions.
Allocation concealment mechanism	16b	Participants were randomly allocated to one of the three experimental groups for an external researcher using EPIDAT 3.1. software (http://www.sergas.es/).
Implementation	16c	Both the participants and the researcher in charge of collecting data were unaware of the assigned intervention and the results obtained, respectively, until the end of the study. An independent researcher using an excel formula, generated a table of random numbers to blind data collectors and outcome adjudicators to ensure an unbiased outcome ascertainment
Blinding (masking)	17a	The randomization sequence was saved on a USB drive and securely stored under lock and key by an independent researcher
	17b	Accessible only when absolutely necessary

Methods: Data collection, management, and analysis

Data collection methods	18a	Instruments employed in the study have reliability and validity. Such us, ODI index is validated to Spanish (32) presenting high sensitivity and specificity to evaluate the function (33,34). The SF-36 measures the quality of life that allows to highlight the satisfactory psychometric properties of internal consistency and the test-retest (35). The VAS scale has shown to have high reliability (α Conbrach= 0.97) [95% CI = 0.96 a 0.98] (36). There are already published studies about diagnosed patients that evaluate their satisfaction using a numeric scale of 11 (-5 to 5) (37,38). TSK Test-retest reliability ranges from r=0.64 to 0.80, and concurrent validity is moderate, ranging from r=0.33 to 0.59 (26). The Spanish version of the Graded Chronic Pain Scale had a high internal consistency (α=0.87) (28). The Spanish version of the PCS has an internal consistency of 0.79 and a test-retest reliability of 0.84 (27).
	18b	Researchers must ensure clear and transparent measurement and reporting of adherence and dropout data. Moreover, individualized treatment will be applied.
Data management	19	The Data Management Plan has been meticulously designed to ensure the quality, integrity, and confidentiality of the data collected during the randomized clinical trial. The study coordinator will assume primary responsibility for data management, performing specific tasks after paper collection. Subsequently, they will create encrypted tables in Excel to ensure participant confidentiality, implementing a double-entry procedure to verify the integrity and accuracy of the records. In the context of study design, essential elements such as the number of patients, inclusion and exclusion criteria, randomization and blinding procedures, treatment and outcome variables have been clearly delineated, all documented in the methodology section of the experimental design. Data collection will be carried out through record forms and electronic systems, with a rigorous policy for eliminating incomplete data to ensure consistency at the study's end.

Statistical methods	20a	Following the Consolidated Standards of Reporting Trials (CONSORT) guidelines on the reporting of RCTs, a per protocol analysis will be performed. The normality assumption will be checked with the Kolmogorov–Smirnov test and box-plot analysis, while the homogeneity of the variance will be tested through the Levene test. To analyze the acute and short effect of SCS and SCS combined with core and control motor exercise on persistent spinal pain syndrome, a two-way ANOVA for repeated measures with experimental groups (i.e., Post3weeks, Post2months, Post6months) as factors will be used, followed by Tukey corrections, to examine time, group, and interaction effects through within- and between-group comparisons (primary and secondary outcomes). Results will be presented as a mean difference (MD) and confident interval at 95% (IC95%). Effect size (ES) will be estimated by calculating Cohen's d coefficient. All analyses will be performed using statistical analysis software SPSS 24 (IBM Inc, Chicago, Illinois, USA).
	20b	Not applicable
	20c	Not applicable
**Methods: Monitoring**		
Data monitoring	21a	"Study Oversight Committee. Composition of the Research Ethics Committee (CEIm) of the Salamanca Health Area President: Head of the General Surgery and Digestive System Service; Vice President: Data Protection Officer of CAUSA; Secretary: Pharmacist and Biochemist; Scientific Committee Representative.<br>IBSAL Members: Clinical Pharmacologist ASCOL President (patient representative) and Clinical Trials Manager
	21b	JVM and FSM investigators will undertake interim analyses, with exclusive access to the outcomes. This approach ensures continuous monitoring of patients and potential adverse effects.
Harms	22	Only the adverse effect of electrode migration in spinal cord stimulation is anticipated. This situation occurs regardless of exercise application. To date, if this happens, the electrode is repositioned if the treatment is effective
Auditing	23	The procedure and frequency for auditing the study will depend on what is established by IBSAL, the institution responsible for the ethics committee, to ensure proper conduct of the study.
**Ethics and dissemination**		

Research ethics approval	24	This study protocol has been approved by the Ethics Committee in research of the Área de Salud de Salamanca (protocol number PI 2023 101435 in (24/01/2024) in accordance with the ethical guidelines of the Helsinki declaration.
Protocol amendments	25	Refinements entail establishing clear strategies or procedures to inform pertinent stakeholders about significant modifications or updates made to the study protocol. This includes continuous communication with participants, research team members, ethics committees, regulatory authorities, and providing detailed explanations of the alterations in study documents or reports throughout the research process.
Consent or assent	26a	The informed consent will be obtained by the nursing team at the hospital. None of them are involved in the development of the experimental study.
	26b	Not applicable
Confidentiality	27	Nursing team at the hospital enrolled participants will be collected, shared, and maintained to protect confidentiality before, during, and after the trial.
Declaration of interests	28	The authors declare that they have no competing interests.
Access to data	29	The principal investigators will have access to the data, and they will analyze the results objectively, having declared no conflicts of interest.
Ancillary and post-trial care	30	There is no financial compensation for participation in the study. The clinical procedure implemented is the one routinely used in everyday clinical practice.
Dissemination policy	31a	All results will be published in scientific journals in the fields of medicine, physiotherapy, and exercise.
	31b	All members of the research team are specialists in exercise and spinal cord stimulation. Furthermore, they are researchers in their respective fields and therefore can contribute to the development of articles in the future publication process
	31c	The protocols implemented regarding treatments will be described to enhance transparency and increase reproducibility. Additionally, the study's results may be provided that there is a justifiable cause, and it is accepted by the authors
**Appendices**		
Informed consent materials	32	Model consent is added. Appendix 1.

Biological specimens	33	Not applicable

*It is strongly recommended that this checklist be read in conjunction with the SPIRIT 2013 Explanation & Elaboration for important clarification on the items. Amendments to the protocol should be tracked and dated. The SPIRIT checklist is copyrighted by the SPIRIT Group under the Creative Commons "Attribution-NonCommercial-NoDerivs 3.0 Unported" license.
